# Case Report: Serum methotrexate monitoring by immunoassay: confusion by by-product, confusion by antidote

**DOI:** 10.3389/fonc.2023.1237178

**Published:** 2023-10-24

**Authors:** Aditya Sharma, Philip Benoit, Frederick Lansigan, David Nierenberg

**Affiliations:** ^1^ Department of Medicine, Dartmouth Health, Lebanon, NH, United States; ^2^ Division of Hematology/Oncology, Dartmouth Cancer Center, Dartmouth Health, Lebanon, NH, United States; ^3^ Department of Pharmacology, Dartmouth Health, Lebanon, NH, United States

**Keywords:** methotrexate, serum, monitoring, antidote, glucarpidase, leucovorin, toxicity

## Abstract

Methotrexate is a commonly used agent in the treatment of many malignancies and rheumatologic/inflammatory diseases. Working by inhibiting dihydrofolate reductase and thereby preventing eventual formation of tetrahydrofolate, methotrexate inhibits synthesis of purines and thymidylate, therefore disabling a malignant cell’s ability to replicate. While it is able to effectively do this, methotrexate also holds potential for significant toxicity. Therefore, serum methotrexate monitoring is of utmost importance when administering the drug, particularly when high doses are used. Although there are several different measurement systems, the immunoassay is a commonly used monitoring system that may be prone to interference when using agents with similar carbon backbone as methotrexate, including folinic acid (leucovorin) at high doses, as well as in the setting of glucarpidase use and consequent methotrexate breakdown. However, adjusting leucovorin dosing policy and being aware of the potential of the immunoassay to be “confused” by similar molecules have allowed for the efficient and effective use of the immunoassay while preventing prolonged hospital stays at our institution.

## 1 Introduction

Methotrexate (MTX), formerly known as amethopterin, was synthesized when folate metabolism and its effect on management of hematologic malignancies were just beginning to be understood. From the summer of 1947, when MTX was first used for management of acute lymphoblastic leukemia (ALL) in pediatric patients, MTX quickly became a mainstay of the anti-metabolite era of malignancy management (1). Although it was first used in the management of ALL in pediatric patients, over the past 70+ years, its clinical utility has extended from treatment of hematologic malignancies to management of autoimmune diseases such as rheumatoid arthritis (2, 3).

Its primary mechanism of action in treatment of malignancy is its ability to reversibly inhibit dihydrofolate reductase, an intracellular enzyme that catalyzes the conversion of the folic acid product dihydrofolate to the active form of folic acid, tetrahydrofolate. In addition, MTX-polyglutamate, a by-product of MTX, has action on dihydrofolate reductase and an effect on thymidylate synthase. MTX is therefore able to decrease the reserve of reduced folates in a cell, functionally inhibiting the synthesis of purines, thymidylate, and methionine and thus decreasing the ability of malignant cells to synthesize DNA and replicate (1).

**Figure 1:**
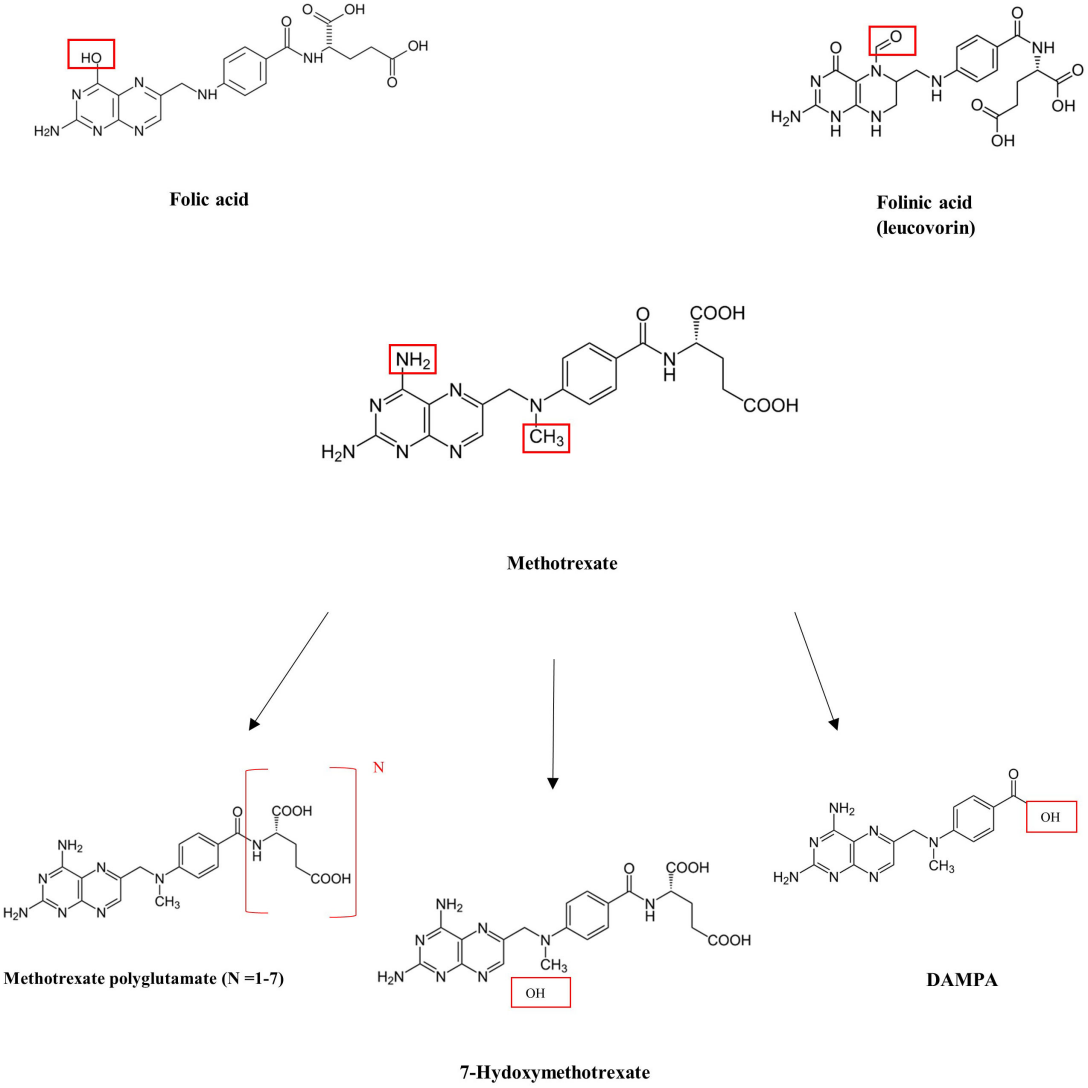
Structures of folic acid methotrexate and derivatives.

MTX is able to inhibit dihydrofolate reductase due to the similar structures of folic acid and MTX (**Figure 1**). With a similar carbon chain backbone and the presence of a glutamic acid group containing two carboxylic acid groups, MTX and folic acid differ only by the presence of a carbonyl group (in folic acid) in place of an amine group (in MTX) as well as the presence of an extra methyl group in MTX. Given the structural similarities, MTX is able to effectively bind and reversibly inhibit dihydrofolate reductase. Its three primary metabolites include 7-hydroxymethotrexate (which is primarily produced in the liver), 2,4-diamino-N10-methylpteroic acid (DAMPA), and glutamic acid, which are primarily produced in the intestines, and methotrexate polyglutamates (MTXPGs), which are primarily produced in red blood cells (4).

While MTX has shown remarkable benefit in the management of many malignancies and autoimmune disease, it is not without risk. MTX plasma levels need to be monitored closely to prevent toxicities as prolonged exposure to high plasma concentrations of MTX can lead to acute kidney injury, myelosuppression, hepatotoxicity, neurologic toxicity, and less commonly pulmonary toxicity and multi-organ failure (5). Of note, MTX is primarily cleared by kidney function, including glomerular filtration and active tubular secretion. Therefore, patients who develop acute kidney injury (AKI), commonly due to crystallization of MTX and 7-hydroxymethotrexate in renal tubules, run the risk of developing severe MTX toxicity as a decline in renal function while MTX would prevent it from being cleared. It is therefore of utmost importance to follow serum MTX levels in order to avoid risk of toxicity, especially when patients receive high-dose MTX (5).

**Figure 2:**
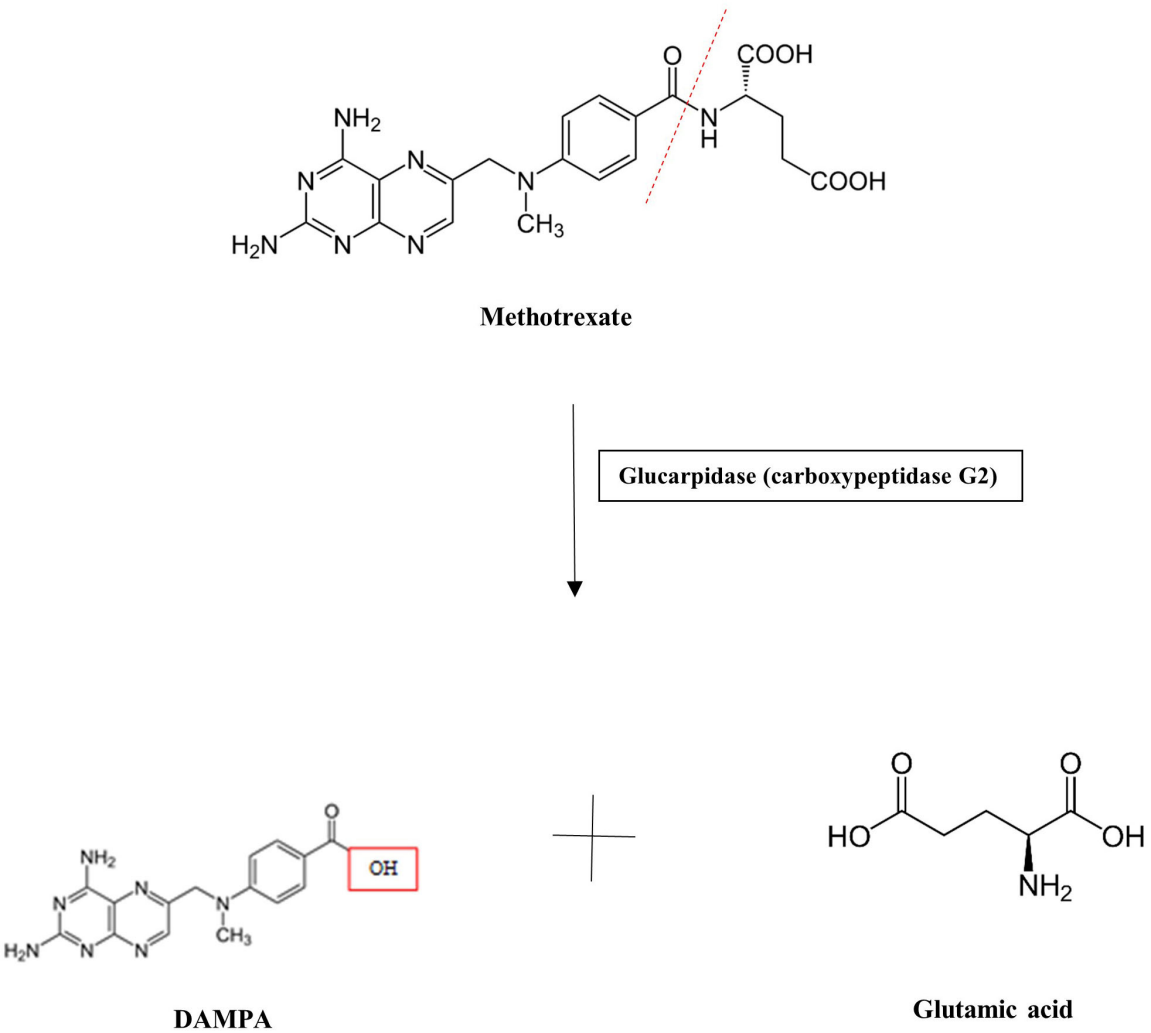
Glucarpidase mechanism of action.

MTX serum or plasma levels have previously been measured via liquid chromatography–mass spectrometry (LC-MS), high-performance liquid chromatography (HPLC), and, more recently, enzyme-linked immunoassays, including the enzyme-multiplied immunoassay (EMIT) (5). While these techniques may aid in drug monitoring, preventative and/or therapeutic action to mitigate toxicity should be initiated if a patient exhibits signs of risk or toxicity. Patients treated with an MTX dose >500 mg/m^2^ (“high dose”) are at risk for toxicity and preventative strategies are warranted. Approaches to the prevention and management of MTX toxicity include hydration with target urine output of at least 100 mL/h, alkalinization of urine to a pH of >7.5 to promote MTX solubility, and, if needed, administration of antidotes to MTX. These antidotes include folinic acid (leucovorin), a reduced form of folic acid similar in structure to both folic acid and MTX, which is able to “rescue” cells by bypassing dihydrofolate reductase. It is able to be taken up by normal-functioning cells due to expression of a reduced folate carrier, whereas malignant cells do not express this carrier and therefore are unable to utilize folinic acid. Additionally, carboxypeptidase G2 (glucarpidase) is an exogenous enzyme that can rapidly metabolize MTX into the inactive by-products normally formed by intestinal metabolism, DAMPA and glutamic acid, and therefore can be used when a patient receiving high-dose MTX demonstrates a serum MTX level >5 μmol/L at 48 h post-dosing (**Figure 2**) (6–10). While leucovorin is often used when using MTX at higher doses for patients with malignancy, glucarpidase is primarily used in the setting of clinical suspicion of MTX toxicity and acute kidney injury, especially following high-dose MTX administration. However, its cost can reach >$100,000 for MTX toxicity rescue dosing and is therefore a limitation of its regular use (7).

As folic acid, MTX, major by-products of MTX, and folinic acid all have similar backbone structures, it is of utmost importance to have accurate and specific measurement systems for therapeutic monitoring. We present two cases in which the commonly used enzyme-multiplied immunoassay (EMIT) system used at our institution appeared to have presented falsely elevated MTX levels, one in the setting of high-dose folinic acid use, and the other following the use of glucarpidase, producing high levels of serum DAMPA and folinic acid.

## 2 Case presentations, diagnostic workup, and outcomes

Case 1

A 69-year-old man with stage IV mantle cell lymphoma presented acutely with symptomatic brain metastases. Given the acute development of neurologic symptoms and imaging strongly suggesting metastatic brain disease, the patient was treated with dexamethasone and then with high-dose MTX (3.5 g/m^2^) for presumed metastatic mantle cell lymphoma. Based on a 24-h post-MTX dosing level of 11.20 μmol/L, his leucovorin dose was increased to 200 mg every 6 h as per institutional policy. Daily MTX monitoring was continued, and the patient demonstrated an expected steady decline in MTX level over the next 3 days. However, over the next 7 days, the MTX levels remained above the 0.10 μmol/L threshold established for safe discharge, and therefore, he remained hospitalized. Transient AKI in the 2 days following MTX infusion was not likely to account for the persistent elevation of MTX level as kidney function had returned to baseline in conjunction with volume expansion and maintenance of a high volume of alkaline urine (pH > 7.5). Investigation for a possible “deep compartment” harboring MTX with slow excretion was undertaken revealing bilateral pleural effusion. Despite drainage of these effusions, serum MTX levels remained above 0.10 μmol/L. In the absence of an alternative explanation, it was hypothesized that the high dose of leucovorin that the patient was receiving (200 mg IV every 6 h) was potentially confounding the EMIT assay used at our institution, and the dose of folinic acid was reduced to 15 mg IV every 6 h on hospital day 12. On hospital day 14, patient’s serum MTX level as measured by the EMIT assay was 0.07 μmol/L, a drop from the previous day’s 0.13 μmol/L.

**Figure 3:**
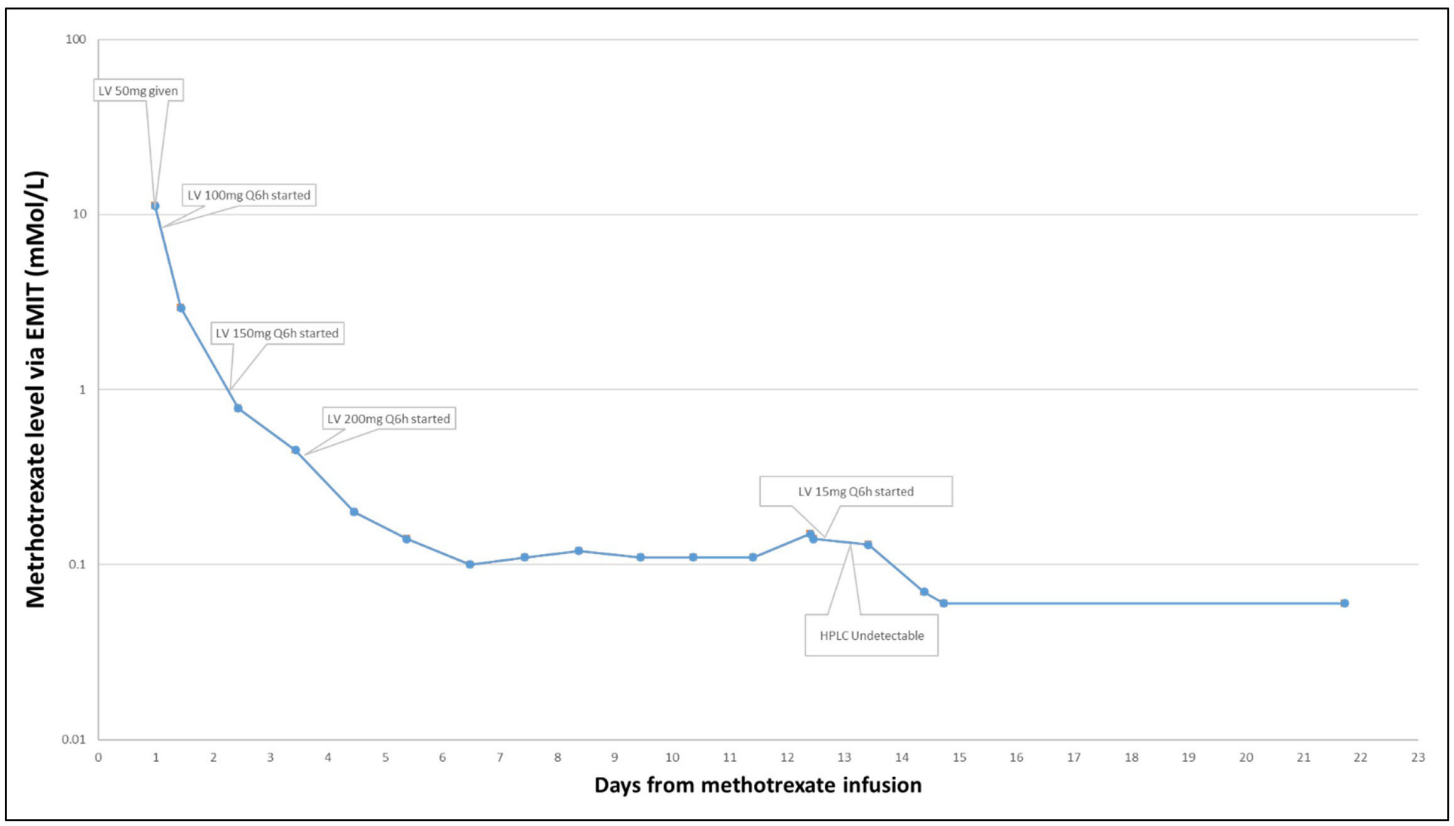
Serum methotrexate levels for Case 1.

To further test this hypothesis, the more specific HPLC assay was run on a blood sample from hospital day 12, when his MTX level on the EMIT assay had been 0.13 μmol/L, which demonstrated an MTX level <0.05 μmol/L, thus further supporting the hypothesis that high doses of IV folinic acid produced high serum levels of folinic acid that confounded the EMIT assay of MTX (**Figure 3**).

Case 2

The patient was a 74-year-old man with a diagnosis of primary CNS lymphoma established by brain biopsy who presented for cycle 6 of rituximab, MTX, vincristine, and procarbazine (R-MVP). He had a history of delayed MTX clearance with previous cycles and was therefore pre-treated with leucovorin 100 mg every 6 h and continuous sodium bicarbonate solution (150 mEq/L) infusion at 150 mL/h with goal urine pH >7.5, and his procarbazine dose was decreased by 25%. He received high-dose MTX (3.5 g/m^2^), following which his kidney function and serum MTX levels were closely monitored with the ARK EMIT assay. Despite maintenance of adequate alkaline urine output, within 48 h of initial MTX dosing, the patient demonstrated AKI, with his creatinine increasing from 0.65 to 1.62 mg/dL. In addition, the patient demonstrated consistently elevated MTX levels as measured by the EMIT assay, with an MTX level 48 h post-dosage of 11.10 μmol/L.

**Figure 4:**
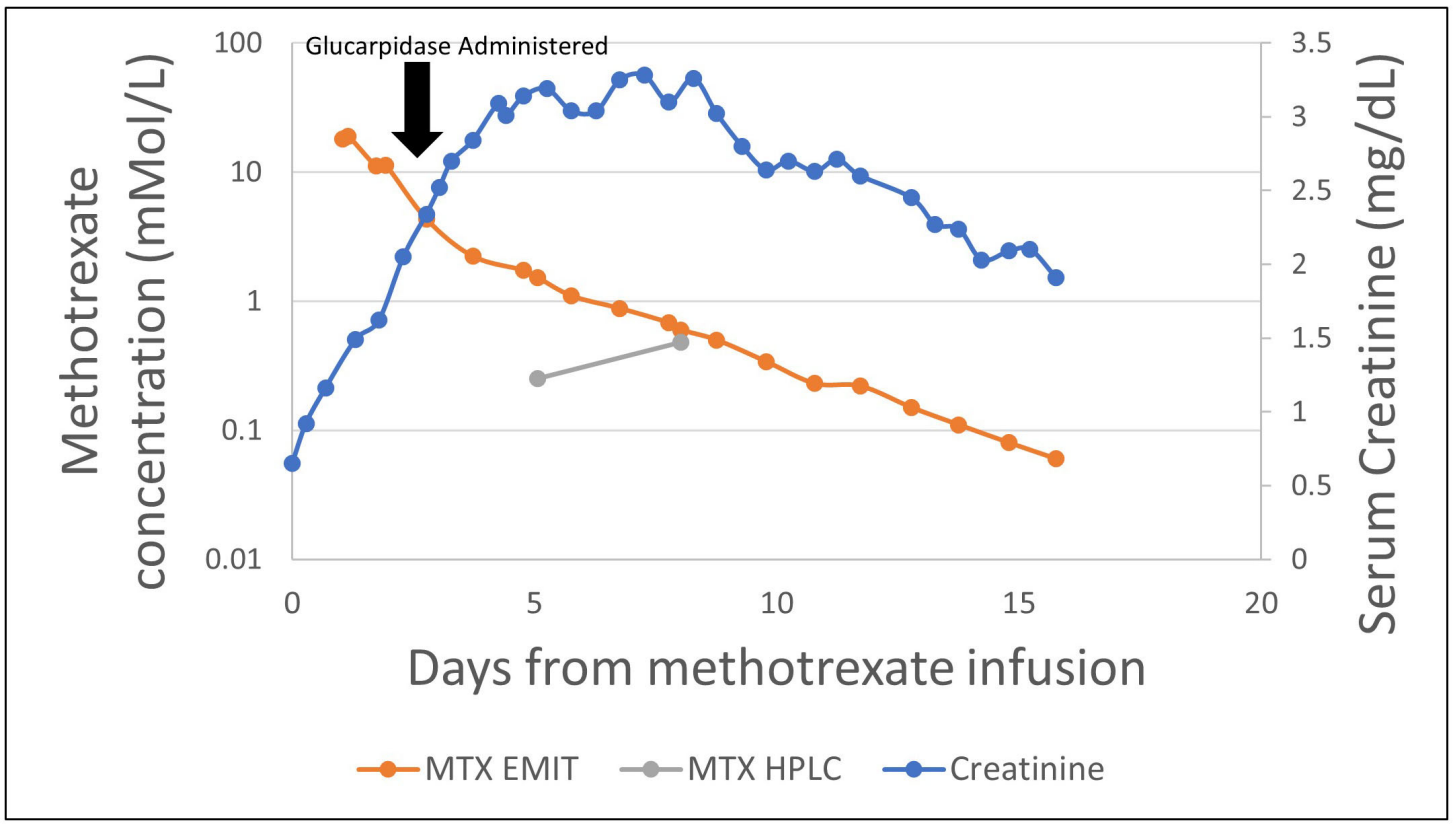
Serum methotrexate levels for Case 2.

Because of AKI and persistent elevation of MTX levels > 5 μmol/L at 48 h post-MTX dosing, he received 3,000 units (50 units/kg) of glucarpidase for rescue from MTX toxicity at 56 h post-MTX. Leucovorin was held 2 h pre- and post-glucarpidase dosing. Workup for a potential reservoir of MTX was unrevealing. Daily MTX levels demonstrated progressive decline over the next 48 h to 1.74 mmol/L by the EMIT assay, but then remained constant at 72 h post-glucarpidase. HPLC assay at the same time (72 h post glucarpidase) demonstrated an MTX level of 0.25 μmol/L. Although another HPLC assay was obtained at 6 days post-glucarpidase dosing demonstrating a high value at 0.48 μmol/L, his MTX levels as per the EMIT assay continued to steadily decline, returning to <0.10 μmol/L at 14 days following glucarpidase dosing (**Figure 4**). It was suspected that this may have represented late redistribution of MTX out of cells and into circulation versus an erroneous result, as his serum MTX levels as per the EMIT assay continued to decline.

## 3 Discussion

MTX monitoring is crucial to patient safety, particularly in the setting of high-dose MTX that is commonly used to treat many malignancies. An accurate and specific assay is crucial to guide management of toxicity and safe discharge (usually considered to be at a level of 0.05 μmol/L or lower). These two cases demonstrate a potential conundrum encountered by clinicians when approaching serum monitoring of HD-MTX, one that calls for a deeper understanding of each method of measurement.

The HPLC method of measurement, as studied by Boer et al., demonstrates a linearity of detection up to 50 μmol/L (*r* ^2^ > 0.99), with a coefficient of variation of <6% for intra-day measurements and <10% for inter-day measurements (11). Furthermore, this method was found to have a lower limit of quantitation, as defined by a coefficient of variation <20%, of 5 nmol/L. In contrast, an iteration of the immunoassay demonstrated linearity of detection with a range of 25–1,000 nmol/L (*r* ^2^ = 0.993) and a greater coefficient of variation of <8% for intra-day measurements and <17% for inter-day measurements. This method was found to have a lower limit of quantitation of 50 nmol/L. Furthermore, it was found that the immunoassay approach demonstrated a consistent positive bias, postulated to be due to interference from folate and MTX metabolites (11). This study suggests not only a higher level of accuracy but also higher levels of precision and decreased rates of positive bias when using HPLC measurements as compared with the immunoassay approach. Similarly, by analyzing 200 clinical plasma samples from children receiving HD-MTX, Albertioni et al. were able to demonstrate that all nonchromatographic methods of measurement were subject to interference from MTX plasma metabolites or endogenous substances, particularly the immunoassay (12).

In Case 1, our patient demonstrated elevated MTX levels by the EMIT assay as compared with the HPLC approach in the setting of receiving high-dose folinic acid, therefore in the setting of folic acid derivatives (folinic acid). In Case 2, our patient received not only high-dose folinic acid, but also glucarpidase, therefore having both folic acid derivatives (folinic acid) and MTX derivatives (DAMPA, 7-hydroxymethotrexate) to potentially confound the immunoassay. Similar to the confounding of serum MTX levels in Case 2, Mulder et al. and Gulley et al. also found serum MTX levels to be falsely elevated when using the immunoassay measurement system following glucarpidase dosing for up to 137 h following glucarpidase administration, with a similar postulation of confounding by MTX metabolites (9, 10).

While the HPLC approach demonstrates a higher level of measuring ability and lower levels of variation in measurement, and is the preferred method of testing, this method is not often as readily available as the immunoassay. In the absence of chromatographic methods of measurement including HPLC and/or LC-MS methods of MTX measurement, alternative solutions may include decreasing the availability of folic acid derivatives, i.e., folinic acid, by decreasing the rescue dosage. Although this change would hypothetically decrease the potential for positive bias, as seen in our limited experience testing decreased rescue doses and demonstrated by Boer, it can also provide potential for MTX toxicity given the lack of availability of reduced folic acid derivatives in the absence of adequate dosing (11). Therefore, further prospective study is required to find the perfect balance between providing effective rescue doses while avoiding the fallout of prolonged hospital courses due to falsely elevated MTX levels, with the goal of improving patient care and judicious use of resources in the process.

## Data availability statement

The original contributions presented in the study are included in the article/supplementary material. Further inquiries can be directed to the corresponding author.

## Ethics statement

Written informed consent was obtained from the individual(s) for the publication of any potentially identifiable images or data included in this article.

## Author contributions

AS contributed to conceptualization, writing, and editing of the manuscript and creation of figures. PB contributed to writing and editing of manuscript and creation of figures. FL contributed to conceptualization, writing, and editing of the manuscript and figures. DN contributed to conceptualization and writing and editing of the manuscript and figures. All authors contributed to the article and approved the submitted version.
